# Predictive Model of The Factors Involved in Cyberbullying of Adolescent Victims

**DOI:** 10.3389/fpsyg.2021.798926

**Published:** 2022-01-05

**Authors:** Ligia Isabel Estrada-Vidal, Amaya Epelde-Larrañaga, Fátima Chacón-Borrego

**Affiliations:** ^1^Department of Research Methods and Diagnosis of Education, Faculty of Education and Sports Sciences of Melilla, University of Granada, Melilla, Spain; ^2^Department of Didactics of Musical, Plastic and Body Expression, Faculty of Education and Sports Sciences of Melilla, University of Granada, Melilla, Spain; ^3^Department of Physical Education and Sports, Faculty of Education Sciences, University of Sevilla, Seville, Spain

**Keywords:** cyberbullying, adolescents, education, behavior, ICT use, peer violence

## Abstract

The development of Information and Communication Technologies has favored access to technological resources in adolescents. These tools provide access to information that can promote learning. However, they can also have a negative effect against people, as they can be used with other functionality, in which cyberbullying situations are caused during the interactions that arise when using social networks. The objective of this study was to determine the predictive value of the role of cyberbullying victims based on variables related to other roles involved in cyberbullying and bullying (aggressors and witnesses), as well as personal characteristics (sex and age), contextual characteristics (type of educational school in which they are attending) and positive teamwork habits. (cooperation, responsibility, dialogue, listening, respect). Information was collected from 227 students of the educational stages of Primary Education and Secondary Education, aged between 11 and 15 years, in a city with a high index of cultural diversity. The step-by-step technique was used to build the regression model. The results indicate that the model has a good goodness of fit coefficient (adjusted *R*^2^: 0.574; *p* < 0.001). The role of cyberbully is the most important predictive variable of the role of the victim in cyberbullying and, to a lesser extent, the role of the witness in cyberbullying, the role of the witness in bullying, and the role of the victim of bullying. The role of the bullying aggressor and the variables sex, age, type of educational center, and teamwork habits are excluded in the predictive model.

## Introduction

Conflict is currently a natural social reality generated by complex situations and interests, ideas, and opinions, which are primarily contradictory between human beings (Silva García, 2008). We also have to count on the innate human condition of people, on the need for power over others, to be supported and loved in the environment in which they move, the desire to possess more and more, and the interest in their survival and wellness (Guillamón, 2019). All these aspects lead to continuous and permanent conflict situations, sometimes reaching extrapolated levels that must be treated as cases of violence. Conflict can be defined as the opposition of interests and/or perceptions and is an essential part of human socialization (Silva García, 2008; Guillamón, 2019; Olejarczyk, 2021).

In the educational field, teachers strive to instill values in students, work on emotional education (Wu et al., 2015), and carry an intercultural education since, as stated by Sánchez and Vargas (2017), an education-oriented toward interculturality is ideal for achieving a respectful and peaceful coexistence and a cohesive society. However, conflicts continue to be frequent in schools and, in many cases, lead to violence between equals in which all students take part, either as aggressors, victims, or witnesses (Garaigordobil and Oñederra, 2010).

Information and Communication Technologies (ICT), mainly the Internet, mobile electronic mail, are advances of the last era which, if used correctly, generate knowledge, culture, and, in general, a global enrichment destined for the entire world society. However, there are people and groups of people such as minors and adolescents, who make inappropriate use of these technologies. In the educational field, many students use them with the intent of harassing and harming their peers (Ortega-Barón, 2017; Garitaonandia et al., 2020).

Cyberbullying, along with bullying, is a social scourge affecting a vital part of society. In most cases, victims of bullying are also reprimanded cybernetically, because with technologies, bullying is transferred to the virtual world (Beltrán-Catalán et al., 2018; de Tejada et al., 2018). Bear in mind that for the attacker, the cyberattack is simpler, faster, and less compromising. It is the model most used by offenders for several reasons: On the one hand, cyberspace is considered the main socialization space, and therefore, all adolescents have a mobile phone and Internet and use it frequently (Garitaonandia et al., 2020). On the other hand, technologies allow the aggressor and the victim not to see each other physically, which prevents seeing the damage caused. In addition, it is possible to maintain the anonymity of the perpetrator, so the aggressor feels protected, which makes it easier for him to commit the aggression (Ortega-Barón et al., 2017; de Tejada et al., 2018; Wang and Ngai, 2020; Barlett et al., 2021; Martínez-Ferrer et al., 2021). Likewise, new technologies allow the grouping of aggressors and the easy and rapid dissemination of audiovisual content. For all that, the damage that is exercised against the victim may be more significant than with bullying (Ortega-Barón et al., 2017). Let us bear in mind that cyberbullying does not have any time or space limitations; it can be carried out in the classroom, in any other school location, like the playground at recess or outside the educational center. The reproduction and dissemination of content can range from environments close to the victim to national or international levels, and the information disseminated is permanently accessible for an extended time (Garaigordobil, 2011; Ortega-Barón, 2017).

Since the cyberbullying plague began, several scientific investigations have been carried out worldwide (Garaigordobil and Oñederra, 2010; Carrascosa et al., 2016; Ortega-Barón et al., 2017; Barlett et al., 2021; Martínez-Ferrer et al., 2021), and with all them, it has been shown that cyberbullying exists among adolescents in all educational centers in the world. There is an average percentage of serious victimization of between 3 and 10%, and between 20 and 30% of adolescents worldwide experience violent behavior, and the most frequent types of harassment are verbal and social exclusion (Garaigordobil, 2011). The organization “Save the Children” has revealed data that confirm that during 2016, 9.3% of adolescents suffered bullying and 6.9% cyberbullying (Nocito, 2017).

There are studies, such as the one by Martínez-Ferrer et al. (2021) that show that girls are more predisposed to participate as victims and boys as aggressors and according to Ortega-Barón et al. (2017), male severe cyberbullying students participate more than girls in the transgression of norms, rejection of institutional authority and direct school violent behavior.

The consequences that cyberbullying causes are serious for all those involved. Although it seems that the victim should be the only one affected, these behaviors also negatively influence aggressors and witnesses since they are at risk of suffering psychosocial and psychopathological imbalances in adolescence and their adult life (Ostrov and Kamper, 2015; Wu et al., 2015; Carrascosa et al., 2016; Sidera et al., 2021). A possible and the most severe consequence of bullying and cyberbullying is suicide, and becoming criminals or aggressors in the relatively near future (Nocito, 2017; Barlett et al., 2021; Martínez-Ferrer et al., 2021).

The victims suffer above all, anxiety, depression, suicidal ideas, fear, low self-esteem, lack of confidence in themselves, feelings of helplessness, nervousness, sleep disorders, and difficulties in concentrating (Carrascosa et al., 2016; Barlett et al., 2021), and according to Núñez et al. (2021), if the level of victimization is high, social anxiety is also high, and self-esteem is low.

The aggressors are morally disconnected from reality (Orue and Calvete, 2019; Wang and Ngai, 2020) and suffer from a lack of empathy, difficulties in complying with the rules, aggressive behavior, criminal behavior, ingestion of alcohol and drugs, technology dependence, and truancy (Garaigordobil, 2011; Ostrov and Kamper, 2015; Carrascosa et al., 2016; Nocito, 2017). As Crone and Konijn (2018) claim, adolescents are sensitive to acceptance and rejection, and if these situations are published on social networks, they become truly emotionally and cognitively vulnerable, which increases the risk of suffering negative consequences after cyberbullying.

In addition, research has been conducted in the field of neurology and neuroscience in which it is claimed that cyberbullying affects the human brain, not only in bullies and victims, but also bystanders (McLoughlin et al., 2020a). Indeed, victims of cyberbullying secrete higher cortisol levels than cyber-aggressors and cyber-witnesses due to stress. Serious cyber-aggressors secrete the least amount of cortisol (González-Cabrera et al., 2017). Furthermore, if the level of victimization suffered during childhood is high, cortisol levels will also be augmented, producing differential effects on brain development in children who are biologically sensitive to stress. Stress influences brain development, and victimization is a stress factor that significantly impacts the brain (Du Plessis et al., 2019; Muetzel et al., 2019). Given this, several researchers have worked with functional magnetic resonance imaging and discovered that viewing images of cyberbullying stimuli activated responses in many regions of the brain, especially those related to social and emotional processing, also the area responsible for feeling self-conscious or to develop empathy (McLoughlin et al., 2020b,c).

On the other hand, educational centers play a significant role in all cases of violence, in the interaction between the parties and must be present in any prevention plan. For this reason, and as a precursor to this work, in 2018, an intervention was carried out in two educational centers of the Autonomous City of Melilla (Spain), one semi-public and the other public, after conducting a pretest questionnaire to a sample of 227 adolescents (Epelde et al., 2019a; Epelde-Larrañaga et al., 2020a,b).

In the pretest, the existence of several cases of bullying and cyberbullying was observed, in forms of verbal aggression (Bullying) and receiving offensive and insulting messages (Cyberbullying; Epelde et al., 2019a; Epelde-Larrañaga et al., 2020a).

Once the data were obtained, a musical intervention was carried out for a duration of 4 months, taking into account the emotional benefits that music brings (Cremades-Andreu and Lage, 2018). Subsequently, the post-test questionnaire gave satisfactory results regarding the decrease in the levels of bullying and cyberbullying in both centers. In the semi-public center, a significant decrease was found in bullying witnesses, cyberbullying victims, cyberbullying aggressors, and cyberbullying witnesses. In the public center, a significant decrease was found in victims of bullying and witnesses of cyberbullying (Epelde-Larrañaga et al., 2020b).

Today there is a great potential for violence and peace, and we believe that everyone must try to stop violence and promote peace, studying the possible peaceful ways to achieve a future without violence.

For this reason, this work gives continuity to those studies mentioned above, predicting the cyberbullying that students may suffer in schools. There are several studies that have worked on the prediction of cyberbullying (Barlett et al., 2017, 2021; Ortega-Barón, 2017; Beltrán-Catalán et al., 2018; Flores et al., 2019; Orue and Calvete, 2019; Wang and Ngai, 2020) and consider that anonymity, insensitive traits, and moral disconnection are predictive attitudes. Directive leaders of the centers and the teaching staff could estimate and prevent potential bullying and cyberbullying situations. We consider that this study is necessary to avoid conflicts and promote coexistence based on respect and tolerance to improve the quality of life of all educational community members.

For this, a multivariable analysis has been carried out as a way to predict cyberbullying in educational centers, following the research background proposed by Martínez-Ferrer et al. (2021); Wang and Ngai (2020); Orue and Calvete (2019); Barlett et al. (2017).

As can be seen, in the problem of cyberbullying, there may be a series of variables that influence the role of the victim, whether they are characteristics of the actors themselves in cyberbullying, or the interaction of the three roles that differ not only in the technological context but also, in the harassment relationships between them from the relationships established in the school environment (Evangelio et al., 2022). Therefore, taking into account all the above, this study intends to advance in the knowledge of the role of the cyber victim in relation to the influence that the various actors in the social networks and outside of them have in the conflict of cyberbullying, thus as variables of the same or contextual. Specifically, the study’s objective was to determine the predictive value of the role of the victim of cyberbullying from variables related to other roles that intervene in cyberbullying and harassment and personal characteristics (sex and age), contextual (type of center), and positive teamwork habits. As a hypothesis, it is postulated that the results will confirm that the role of the victim in cyberbullying can be predicted by all the variables mentioned above.

## Materials and Methods

### Contextualization

The research was carried out in two centers in the autonomous city of Melilla (Spain), located in North Africa on the shores of the Mediterranean Sea. The population is 86,487 inhabitants (Instituto Nacional de Estadística, 2019) is of great interest for researchers because it presents cultural wealth, the result of their geographical position and coexistence of different religions (Christians, Muslims, Jews, Hindus, and Roma), cultural origins (European, Riff, Eastern) and mother tongues (Spanish, Tamazight or Israeli) since the 19th century. Due to the city’s strategic location, it is considered a “gateway to Europe” in which there is a large influx of immigrants whose children attend school during the period of residence in Melilla. There are 15 Primary Education centers (12 public education and two concerted) and eight Secondary Education centers (six public education and two concerted). However, the local educational administration does not allow the collection of information differentiating cultural characteristics, which is why the present study shows the data within a multicultural context without carrying out analyses that rely on the predictive factors in each culture.

### Design and Participants

The population under study comprises all students enrolled in 6th grade (last year of Primary Education) and 7th grade (first year of Secondary Education) in the Autonomous City of Melilla. A representative sample of 227 schoolchildren was established (sampling error of 0.05; CI = 90%). There is a slightly higher proportion of female students (54.6%) than male students (45.4%), who studied in the first year of Secondary Education (73.6%) and the last year of Primary Education (26.4%).

**Figure 1:**
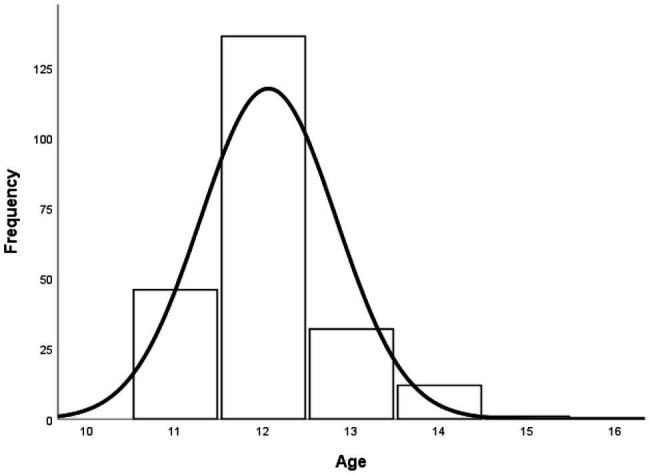
Distribution of students by age.

The mean age is 12.06 years (*SD* = 0.771), the youngest participant was 11 years old, and the oldest was 15. In Figure 1, it can be seen that the highest percentage of students corresponds to those who are 12 years old (59.9%), followed by 11 years (20.3%), 13 years (14.1%), 14 years (5.3%), and 15 years (0.4%).

### Instruments

Two instruments have been used. Cyberbullying: Screening of harassment between equals (Garaigordóbil, 2013) and Teamwork Test, a self-elaborated questionnaire validated by a panel of experts (Epelde et al., 2019b). The first instrument has two sections. The first collects information on bullying through 12 items, distinguishing four types of face-to-face bullying (physical, verbal, social, and psychological) grouped according to the role of the bullying situation (victim, aggressor, and observer), responding from the three roles in a triangular way. The second section refers to cyberbullying through 45 items, in which 15 types of behavior are evaluated, answered from the three mentioned roles. The responses follow the four-option Likert-type scale (0 = nothing to 3 = always). The sum of the scores obtained in each of the sections allows the classification of the various roles. The instrument’s internal consistency is good, with a Cronbach’s alpha coefficient of 0.81 for the bullying section and 0.91 for the cyberbullying section (Garaigordóbil, 2013).

The Teamwork Test is composed of 14 items that score the ability to work in a group of school adolescents (e.g., “You contribute to a pleasant work environment”). The evaluations follow the Likert-type scale of four options (1 = never to 4 = always). The instrument’s internal consistency, measured with Cronbach’s alpha, is acceptable (*α* = 0.806; Epelde et al., 2019b).

### Procedure

The participation of the sample was requested through a meeting held between the Department of Didactics of Musical, Plastic and Body Expression of the University of Granada, with the management team of several schools. Topics on the nature and objective of the study were discussed. Subsequently, the informed consent of the legal guardians of the adolescents was requested in those educational centers that decided to participate in the research. To do this, they signed an authorization model to collect information from the teenagers. Regarding the application of the instruments, it was carried out during school hours in the presence of the researchers to ensure their correct application. The anonymity of all students was ensured, who participated voluntarily, and was conducted according to the guidelines of the Declaration of Helsinki.

### Data Analysis

The stepwise multiple linear regression multivariate analysis technique was applied to establish how certain predictive or explanatory variables are related to the criterion variable. Specifically, the predictive value of the role of victim in cyberbullying was calculated from the role of the variables of the aggressor in cyberbullying, the role of witnesses in cyberbullying, the role of the victim in bullying, the role of the aggressor in bullying, the role of the witnesses in the harassment, sex and age of the students, type of center in which they are schooled and the positive habits of teamwork they have. The analyses allow knowing the model’s assumptions except for homoscedasticity, so it was checked with the Leven test to know if *p* > 0.05. Statistical analysis was performed using the IBM SPSS^®^ 25.0 statistical software (International Business Machines Corporation, Armonk, NY, United States).

## Results

**Figure 2:**
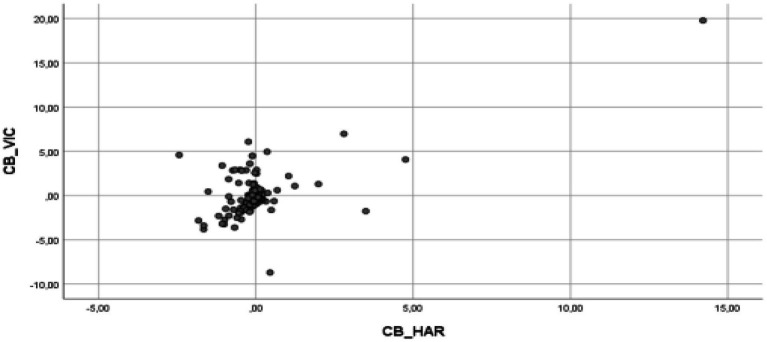
Partial regression graphs of the cyberbullying role of victims criterion variable (CB_VIC), with the cyberbullying role of the bully predictive variable (CB_HAR), included in the model.

**Figure 3:**
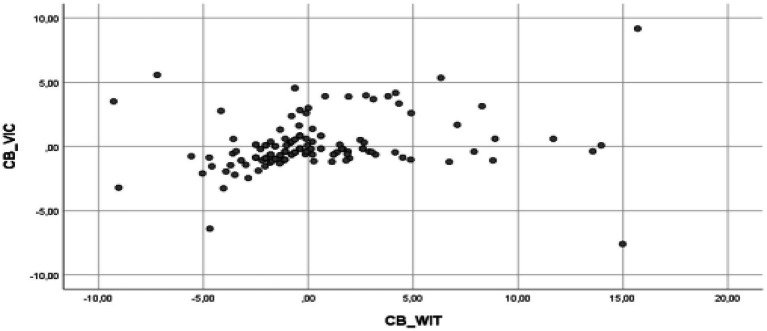
Partial regression graphs of the cyberbullying role of victims criterion variable (CB_VIC), with the cyberbullying role of witnesses predictive variable (CB_WIT), included in the model.

**Figure 4:**
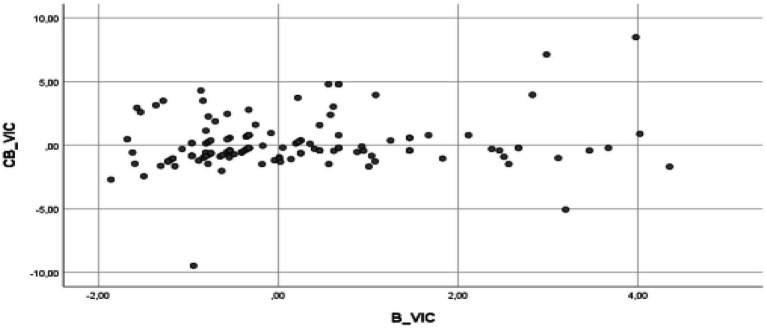
Partial regression graphs of the cyberbullying role of victims criterion variable (CB_VIC), with the predictive variable the role of harassment of victims (B_VIC), included in the model.

**Figure 5:**
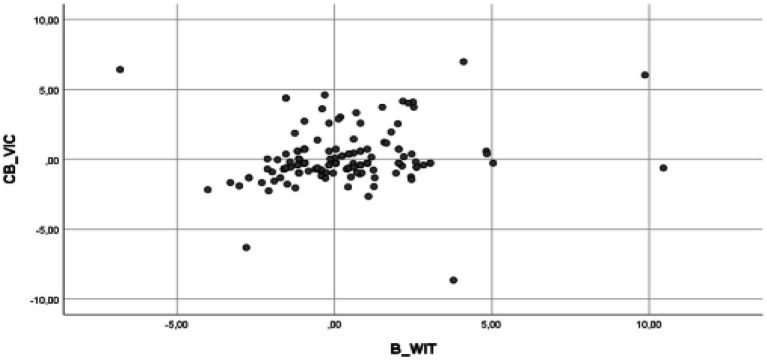
Partial regression graphs of the cyberbullying role of victims criterion variable (CB_VIC), with the predictive variable the role of harassment of witnesses (B_WIT), included in the model.

**Figure 6:**
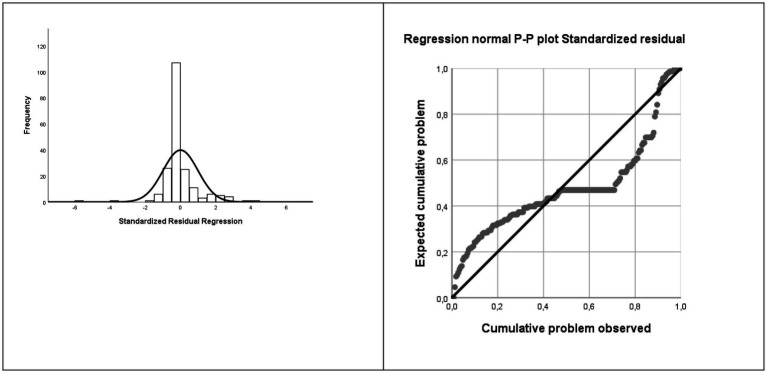
Normality assumption: Histogram and Normal probability graph of the criterion variable.

The multiple linear regression analysis results indicate that the assumptions of the model are fulfilled, and therefore, validating the model (Aragón et al., 2015). Thus, it has been verified that it complies with the assumption of the model (Vilá et al., 2019), specifically, the assumption of linearity (see Figures 2–5 for the partial dispersion diagrams), independence of the errors (Durbin-Watson is between 1.5 and 2.5; Table 1), normality (Figure 6), homoscedasticity (*p* > 0.05), non-collinearity (tolerance greater than 0.10, and inflation factor of the variance less than 0.10; Table 2).

**Table 1:** Stepwise Multiple Linear Regression Model.

Model^e^	*R*	*R* ^2^	*R*^2^ adjusted	Standard error estimate	g.l.	*F*	p de F	Durbin-Watson
1	0.689^a^	0.475	0.472	1.86199	1	177.435	0.001	
2	0.738^b^	0.545	0.540	1.73878	2	116.618	0.001	
3	0.754^c^	0.569	0.562	1.69587	3	85.393	0.001	
4	0.763^d^	0.582	0.574	1.67356	4	67.315	0.001	1.749

a Predictive variables: (Constant), Cyberbullying aggressors.

b Predictive variables: (Constant), Cyberbullying aggressors, Bullying witnesses.

c Predictive variables: (Constant), Cyberbullying aggressors, Bullying witnesses, Cyberbullying witnesses.

d Predictive variables: (Constant), Cyberbullying aggressors, Bullying witnesses, Cyberbullying witnesses, Bullying victims.

e Dependent variable: Cyberbullying victims.

**Table 2:** Coefficients in the Multiple Linear Regression Model equation.

Model		Non-standardized coefficients		Standardized coefficients	*t*	*p*	Collinearity statistics	
		*β*	Standard error	*β*			Tolerance	VIF
4^a^	(Constant)	0.129	0.157		0.822	0.412		
	Aggressor role in Cyberbullying	1.181	0.099	0.592	11.948	0.001	0.880	1.136
	Witness role in Bullying	0.153	0.062	0.137	2.452	0.015	0.690	1.449
	Witness role in Cyberbullying	0.109	0.035	0.174	3.148	0.002	0.710	1.408
	Victim role in Bullying	0.259	0.104	0.130	2.491	0.014	0.790	1.265

a Dependent variable: Role of the cyberbullying victim.

The multiple linear regression analysis suggested four models, with model 4 offering the most significant explanatory capacity (Table 1). The goodness of fit of the model can be considered adequate. Specifically, 57.4% of the variance in the role of the victim in cyberbullying is explained by the four variables introduced in the model: role of the aggressor in cyberbullying, the role of the witness in cyberbullying, the role of the victim in bullying, and the role of the witness in the bullying. Therefore, the role of the aggressor in bullying, the sex and age of the students, the type of center in which they are enrolled and positive teamwork habits are excluded.

Concerning the selected predictive model, Table 2 shows that the value of t is associated with a probability of error of less than 0.05 in the four variables included in the model. Likewise, the standardized coefficients indicate which variables have a greater explanatory weight in the model. The role of the bully in cyberbullying is the strongest predictor to predict the role of the victim in cyberbullying (*β* = 0.592). To a lesser extent, it is followed by the role of the witness in cyberbullying (*β* = 0.174), the role of the witness in bullying (*β* = 0.137), and the role of the victim in bullying (*β* = 0.130).

Therefore, an adolescent will receive more harassment in a virtual environment when there is a tendency from other adolescents to harass him in the same environment (aggressor role). Such harassment slightly increases if there are virtual witnesses to cyberbullying. In addition, the cyberbullying suffered by the victim increases if there have been witnesses in the case of having suffered harassment in the face-to-face environment. Consequently, although to a lesser degree, the harassment received by the victim also increases when he is harassed on other occasions in the face-to-face environment.

Finally, the predictive equation reached with the multiple linear regression analysis is shown below if used in particular contexts. This equation makes it possible to predict the role of an adolescent as a victim in a cyberbullying situation based on the scores obtained by two classmates, one acting as an aggressor and the other as a witness. The predictive equation would be the following:


$${CB}_{Vic}=0.129+0.592\;{CB}_{Agr}+0.174\;{CB}_{\mathrm{Wit}}+0.137\;B_{\mathrm{Wit}}+0.130\;B_{Vic}$$

Where CB_Vic_ = score that an adolescent would have from the role of victim in a cyberbullying situation; 0.129 = constant of the equation; CB_Agr_ = score obtained by another adolescent in the role of aggressor in cyberbullying; CB_Wit_ and B_Wit_ = score obtained by another adolescent who acts as a witness in the situation of cyberbullying and bullying, respectively; and B_Vic_ = score obtained by the adolescent who acts as a victim outside the technological environment (bullying situation).

## Discussion

Considering the results obtained according to the objective of the study “to determine the predictive value of the role of the victim of cyberbullying from variables related to other roles that intervene in cyberbullying and bullying, as well as personal characteristics (sex and age), contextual (type of center in which they are enrolled) and positive teamwork habits,” the predictive variables of the role of the victim in cyberbullying are the role of the aggressor in cyberbullying, the role of witness in cyberbullying and bullying and the role of the victim in bullying. However, the role of the aggressor in bullying and the variables sex, age, type of center, and capacity for teamwork remain out of the equation. Therefore, in response to the hypothesis raised at the beginning, it has been found that the role of the victim cannot be predicted by all the proposed variables but by the four predictor variables not excluded from the model.

Until now, regarding the predictor variables related to the different roles in cyberbullying, numerous studies have identified the relationship between victims and the existence of aggressors in both traditional bullying and cyberbullying; however, this study advances toward a greater understanding of the effect of the other roles present in both manifestations of school violence, such as witnesses and victims of bullying.

The role of cyberbully is the one that has a greater weight in the prediction, coinciding with different studies such as those of Martínez-Ferrer et al. (2021). However, the role of the bullying aggressor does not intervene to explain victimization in cyberbullying. This result differs from those reported in other studies (Juvonen and Gross, 2008; Waasdorp and Bradshaw, 2015; Baldry et al., 2016; Kim et al., 2017; Khong et al., 2020) in which it is observed that schoolchildren who display aggressive behaviors in face-to-face bullying also exhibits them online, and there is a greater probability of suffering cyberbullying among people who have been or are victims of traditional bullying. Therefore, the results obtained in this work would support the results of Orue and Calvete (2019) and Fanti and Kimonis (2013), in which a differentiation is identified between the behaviors of the aggressors in bullying and cyberbullying. This observation leads to creating a cyberbully profile in which insensitivity and moral disconnect traits prevail over those related to impulsivity and feelings of grandiosity that predominate in traditional bullying aggressors. This differentiation can also be supported by the anonymity of cyberbullying and, therefore, by the non-need for specific traits to be present in the role of the aggressor, such as those specified by Lucas et al. (2011) and Cerezo (2009). However, according to the work of Wang and Ngai (2020), anonymity has an indirect effect on the prediction of cyberbullying being more closely associated with moral disconnection.

Regarding the predictive value of the victim in cyberbullying based on the role of the victim of bullying, although we observed an attenuated effect compared to the influence exerted by the cyberbully, it is consistent with the results obtained in the investigations of Del Rey et al. (2012) and Lazuras et al. (2017), and with the previously cited works on continuity and overlap in the roles of both aggression and victimization (Estévez et al., 2020).

Witnesses to cyberbullying and bullying also have an explanatory effect on the role of the cyber victim. Although there is no study based on multivariate analysis to visualize this interaction, numerous studies specify the need to carry out action programs that include all active or passive participants in bullying situations [ConRed programs by Ortega-Ruiz et al., 2012; ViSC by Solomontos-Kountouri et al., 2016 or Cyberprogram 2.0 by Garaigordobil and Martínez-Valderrey, 2016]. Victims in both Primary and Secondary Education suffer consequences in their physical and psychological health, among which is having feelings of loneliness, dissatisfaction, and distrust in the environment due to their low social acceptance or loss of control of their social relationships (Cerezo, 2009; Lucas et al., 2011), this may be associated with the perception of lack of social support from their peers. In this sense, it is necessary to carry out specific interventions to promote active attitudes toward detecting harassment, thus reducing the perpetuation of situations of harassment through inaction.

The variables sex, age, and type of center have been excluded from the obtained predictive model. Concerning sex, these results are not in line with those of Martínez-Ferrer et al. (2021), in which sex has a more moderating effect on cyber-victimization and cyberbullying in boys than in girls. Even though other studies reported a relationship between sex and victimization, more associated with the female gender, fundamentally in the secondary education stage (Selkie et al., 2016; Wolke et al., 2017), and sex and aggression, more associated with the male gender (Avilés, 2009; Calvete et al., 2010; Del Rey et al., 2012), this association has not had enough of an effect on the prediction of the role of the cyber victim in the present work.

No studies have been found in which the effect of age on the prediction of cyberbullying is analyzed, taking into account that the present work focuses on the last year of Primary Education and the first year of Secondary, and most of the studies focus on the Secondary Education stage as confirmed by Sidera et al. (2021). Although descriptive studies indicate that bullying is higher in private and semi-public centers (León et al., 2011), we did not observe this effect. It is plausible that not including a representative sample of the different types of educational centers would reveal the effect that it may have on the variables analyzed.

The limitations of our study are related to the sample size. A more extensive data set would have allowed us to investigate the influence of the center type in which the students are enrolled and positive teamwork habits. Likewise, there has been a lack of investigations that analyze cyberbullying and specifically those of an explanatory type, in addition to a substantial dispersion of research instruments to analyze the involvement in cyberbullying with which to compare the results obtained. It has also been identified that most studies focus on students of Compulsory Secondary Education and a minority include the age group 10 to 12 years, even though according to Garaigordobil and Oñederra (2008), it is the age with the most significant presence of these kinds of behaviors. The primary education stage requires the completion of specific studies, taking into account that the incidence of cyberbullying and its repercussions from the psycho-affective point of view is different with respect to older schoolchildren (Sidera et al., 2021).

According to the results, the relationships between harassment and cyberbullying environments can predict the participant agents’ behavior. Thus, in the case that concerns us, an adolescent who suffers bullying (victim) in a virtual environment has a greater tendency to suffer it when the aggressor in the said environment is more active. Although the presence of witnesses both on social networks and in face-to-face places also affects them, the harassment that the victim receives outside of the technologies is not as decisive as the role played by the aggressor.

Therefore, future research seeking to identify which factors cause an aggressor to be more active within social networks, since it affects the victim within the virtual environment, and which factors predict the aggressor’s level of activity in face-to-face settings are essential. Also, we consider it necessary to delve into the existence and characteristics of the two types of aggressors, the aggressors active in bullying and cyberbullying and the aggressors active only in cyberbullying. It is also necessary to expand the studies in which the effect of observers in the prevention of bullying is analyzed and include other variables such as those that analyze behaviors in social networks and other types of communication tools with technological support in which the behaviors occur of violence among schoolchildren.

Furthermore, as a practical application, it is also necessary to continue implementing prevention and intervention programs with schoolchildren and their families, promoting an education based on coexistence and respect for others, together with specific actions on each of the roles of cyberbullying, aimed primarily, at improving emotional intelligence and social skills at an early age. This knowledge would help the actors understand and empathize with bullying situations (Inglés et al., 2014; Nocito, 2017), motivating them to reduce the promotion of said relationships.

The main action for the aggressors is to make them aware of the negative impact that their actions have on adolescents, whatever their environment of action, reinforcing and improving their sensitivity and moral values (Cuadrado-Gordillo and Fernández-Antelo, 2019; Orue and Calvete, 2019).

This awareness-raising work can lead to a real and effective improvement of coexistence and strengthen the values on which it is based, which will lead to a more peaceful, more caring, more humane society both in the educational field and outside it. Even though human relations and coexistence have suffered an evident deterioration in educational center, humans have enormous potential to reverse the situation.

## Conclusion

The results obtained in the study allow the following conclusions to be drawn:

The aggressor’s behavior on social networks is what really defines the degree of harassment of the victim. The profile of the cyber-aggressor is characterized by his callousness and moral disconnection. Adolescents receive more cyberbullying when the aggressor is more active, and when other adolescents are witnesses, especially in virtual environments. Additionally, the degree of virtual harassment increases slightly in case of being a victim in face-to-face environments. The victim’s behavior in cyberbullying is not influenced by the aggressor’s behavior in face-to-face situations, nor by sex, age, type of center, and teamwork habits, because they were excluded in the predictive model.

For all these reasons, it is recommended to develop educational intervention programs aimed at students that allow them to acquire coping strategies in cyberbullying situations in the case of victimization, especially in the face of the behavior of the aggressor and witnesses in the said environment. Among the strategies are assertiveness, emotional intelligence, self-regulation, or an adequate use of personal information in virtual environments. In the same way, it would be convenient to train teachers and families to identify signs that can occur in adolescents as signs that make the bullying situation visible in order to help them. Even the specialized training of teachers would allow students to guide them in developing these coping strategies. Likewise, students must be made aware of the feelings that the victim provokes in a bullying situation, and of the situational reality that is provoked. It is of special interest to intervene in pre-adolescents to favor the prevention of bullying situations.

## Data Availability Statement

The original contributions presented in the study are included in the article/supplementary material, and further inquiries can be directed to the corresponding author.

## Ethics Statement

Ethical review and approval were not required for the study on human participants in accordance with the local legislation and institutional requirements. Written informed consent to participate in this study was provided by the participants’ legal guardian/next of kin.

## Author Contributions

AE-L and FC-B: conceptualization. AE-L and LE-V: methodology. LE-V: formal analysis. All authors contributed to the interpretation of the data and writing – original draft preparation. All authors have written – review and edited. All authors have approved the final manuscript.

## Conflict of Interest

The authors declare that the research was conducted without any commercial or financial relationships that could be construed as a potential conflict of interest.

## Publisher’s Note

All claims expressed in this article are solely those of the authors and do not necessarily represent those of their affiliated organizations, or those of the publisher, the editors and the reviewers. Any product that may be evaluated in this article, or claim that may be made by its manufacturer, is not guaranteed or endorsed by the publisher.
